# LATENT CLASSES OF RESILIENCE IN A NATIONWIDE SAMPLE OF AMERICAN ADULTS DURING COVID-19 PANDEMIC: THE ROLE OF AGE

**DOI:** 10.1093/geroni/igad104.2052

**Published:** 2023-12-21

**Authors:** Joyline Chepkorir, Melissa Hladek, Jing Huang, Noelle Pavlovic, Phillip Phan, Alden Gross

**Affiliations:** Johns Hopkins University, Baltimore, Maryland, United States; Johns Hopkins University, Baltimore, Maryland, United States; Johns Hopkins University, Baltimore, Maryland, United States; Johns Hopkins School of Nursing, Baltimore, Maryland, United States; Johns Hopkins University, Baltimore, Maryland, United States; Johns Hopkins University Bloomberg School of Public Health, Baltimore, Maryland, United States

## Abstract

Intra-individual coping and responses to stressors vary across people, influencing health outcomes. We aimed to identify distinct subgroups, using latent class analysis, of resilience based on coping resources and responses to COVID-19 using data from a nationally representative sample of American adults (N=3,640), including 2,507 younger (age< 65 years) and 833 older adults (age≥65 years). Data were collected in June 2020, after the first three months of COVID-19 lockdown. We identified sub-groups of resilience based on 11 self-reported coping resources and responses: pain, trauma, depressive and anxiety symptoms, alcohol, drug and food usage, coping resources, social networks, perceived cognitive deficit, and loneliness. Using conventional fit criteria (AIC, BIC, LMR-LRT, BLRT), we identified four latent classes: Low mental/physical resilience (N=187, 5.6%), Low mental resilience (N=432, 13.0%), Low social resilience (N=832, 25.0%), and High resilience (N=1888, 57%). While the first resilience class showed the poorest coping in all measures, the fourth class displayed good resilience qualities across all responses. Low mental resilience was characterized by high anxiety and depression, while the Low social resilience class was characterized by relatively poor social networks. Adults in the High Resilience class were on average 54 years old while adults in the low resilience class were considerably younger (average 33 years old, p< 0.001). Understanding patterns of resilience, especially by age strata, may help identify and support those who have an increased risk for poorer outcomes and may guide the development of individual- and population-level interventions to promote adaptive coping in the event of such a global stressor.

